# Sweet Quinine and Svapina

**Published:** 1869-01

**Authors:** 


SWEET QUININE AND SVAPINA.—We have received from Frederick Stearns of Detroit, samples of Sweet Quinine and Svapina, medicines which will at once attract the attention of physicians.

The quinine has very little of the bitter taste of the drug, and possesses on this account decided superiority over all other preparations. To obtain the same effects, it is probable that a little increase in the dose would be necessary.

The Svapina is said to contain only the anodyne and soporific alkaloids of opium, codeia, narceia and morphia, and to exclude all others. We have not had opportunity to test its action, but have no doubt it will be found reliable as an anodyne, possessing advantages where the ordinary preparations of opium act unfavorably.

